# Dietary supplementation with multi-strain formula of probiotics modulates inflammatory and immunological markers in apical periodontitis

**DOI:** 10.1590/1678-7757-2020-0483

**Published:** 2021-01-25

**Authors:** Leopoldo COSME-SILVA, Renan DAL-FABBRO, Luciano Tavares Angelo CINTRA, Edilson ERVOLINO, Alana Sant’Ana do PRADO, Daniel Pinto de OLIVEIRA, Priscylla Gonçalves Correia Leite de MARCELOS, João Eduardo GOMES-FILHO

**Affiliations:** 1 Universidade Federal de Alagoas Faculdade de Odontologia Departamento de Odontologia Restauradora Maceió Brasil Universidade Federal de Alagoas (UFAL), Faculdade de Odontologia , Departamento de Odontologia Restauradora , Maceió , Brasil .; 2 Universidade Estadual Paulista Faculdade de Odontologia de Araçatuba Departamento de Odontologia Preventiva e Restauradora Araçatuba Brasil Universidade Estadual Paulista (UNESP), Faculdade de Odontologia de Araçatuba , Departamento de Odontologia Preventiva e Restauradora , Araçatuba , Brasil .; 3 Universidade Estadual Paulista Faculdade de Odontologia de Araçatuba Departamento de Ciências Básicas Araraquara Brasil Universidade Estadual Paulista (UNESP), Faculdade de Odontologia de Araçatuba , Departamento de Ciências Básicas , Araraquara , Brasil .

**Keywords:** Apical periodontitis, Inflammatory infiltrate, Interleukin, Bone resorption, Probiotics

## Abstract

**Objective:**

The aim of this study was to evaluate whether probiotics multi-strain formula affects the development of apical periodontitis (AP) induced in rats.

**Methodology:**

16 Wistar rats were divided in two groups (n=8): rats with AP fed with regular diet (Control-C (CG)); rats with AP, fed with regular diet and supplemented with multi-strain formula (one billion colony-forming units (CFU)): GNC Probiotic Complex (PCG) ( *Lactobacillus acidophilus, Lactobacillus salivaris, Lactobacillus plantarum, Lactobacillus rhamnosus, Bifidobacterium bifidum, Bifidobacterium animalis* subs. lactis and *Streptococcus thermofilus* ). AP was induced in the upper and lower first molars by dental pulp exposure to the oral environment. PCG was administered orally through gavage for 30 days during the AP development. After this period the animals were euthanized and the mandibles were removed and processed for histologic analysis, and immunochemical assays for interleukin (IL)-6, IL-10, IL-1β, RANKL, OPG, and TRAP. The Mann–Whitney U test and Student’s t test were performed (P<.05).

**Results:**

The CG showed more intense inflammatory infiltrate than the PCG group (P<.05). IL-1β, IL 6 and RANKL decreased in the PCG group compared with CG (P<.05). The IL-10 level increased in the PCG group (P<.05). The OPG level was similar in both groups (P>.05). The number of mature osteoclasts (TRAP-positive multinucleated cells) was lower in PCG group when compared to the CG (P<.05).

**Conclusion:**

Probiotic Complex modulates inflammation and bone resorption in apical periodontitis.

## Introduction

Despite recent modernization and mechanization in endodontic therapy, conventional treatments for periaptical periodontitis still demand new adjunctive therapies. However, beneficial microorganisms has arisen as a potential concept. According to the World Health Organization, probiotics are “live microorganisms that can confer health benefits to the host when consumed in adequate amounts”, modulating host immunoinflammatory response and influencing the structure and function of microbiota. ^1^ On the other hand, the concept of probiotics use in addressing endodontic disease is new and has not been studied adequately. ^2^

Recently, several studies assessing the effects of probiotics on periodontal disease showed encouraging results regarding the properties of these beneficial strains. ^3 , 4^ In an investigation, patients with chronic periodontitis were selected and received *Bifidobacterium animalis subsp. lactis* as supplementary treatment (probiotic group). This group presented a decrease in probing pocket depth and a clinical attachment gain significantly higher than the control group. ^4^ Also, the probiotic group displayed lower proinflammatory cytokine levels when compared to the control group, proving that this supplementation promotes additional clinical, microbiological, and immunological benefits in the treatment of chronic periodontitis. ^3^ In endodontics, a preliminary study indicated that probiotic organisms of the species Lactobacillus and Bifidobacterium are effective for preventing the growth of *E. faecalis* and *C. albicans in vitro* . ^2^

Chronic periodontitis and apical periodontitis are highly prevalent oral diseases in the world population. ^5 , 6^ The pathogenic process of apical periodontitis can be initiated by an injury (usually bacteria) evoking an acute immune-inflammatory host response in the periapical region. Once the immune system cannot eliminate the causative agent itself, a succession of events occurs to restrict the infected area (forming a circumscribed barrier) and prevent its spread. ^5 - 7^

The effect of probiotics on the immune response has been well investigated. ^8 - 12^ Much of these investigations in animal and human models suggest that probiotics may stimulate both nonspecific and specific immune responses. These positive effects on the immune system occur without triggering a harmful and potentially inflammatory response, being mediated by macrophages activation, an increase in anti-inflammatory cytokine levels, an increase in natural killer cell activity and an increase in immunoglobulin levels. ^8 - 12^

It has been shown that, in isolation, probiotics *Lactobacillus rhamnosus* and *Lactobacillus acidophilus* had a significant effect on inflammation reduction and bone resorption in apical periodontitis development in rats. ^9 , 13^ However, the immune response can be increased when one or more probiotics are administered **in association** and act synergistically, as appears to be the case with *Lactobacillus* administered **in association** with *Bifidobacterium* . ^14 , 15^ Nowadays, although some studies are suggesting the use of single-strain probiotics in endodontics, ^9 , 13^ none of the studies addresses the most common commercial form in the world: the administration of probiotics through mixtures of strains. ^1 , 14 , 15^ Especially when in association with mixed strains result in an additive or even synergistic outcomes in terms of bioactivity by the component strains. ^1 , 14 , 15^

To the best of our knowledge, no investigations assessed the effects of a probiotic complex on the apical periodontitis development. Therefore, the purpose of this study was to evaluate the histopathological and immunohistochemical outcomes following the administration of the probiotics multi-strain formula (GNC Probiotic Complex CODE424639) in rats with induced apical periodontitis. The null hypothesis tested was that there would be no difference between the no probiotics group (CG) and the multi-strain probiotics formula group (PCG).

## Methodology

### Animals

Sixteen male Wistar rats ( *Rattus norvegicus albinus* ), each weighing 200-250 g, were used in this study. The animals were housed in a temperature-controlled environment (22°C±1°C, 70% humidity) with a 12 h light-dark cycle and *ad libitum* access to water and food. ^9 , 13 , 16^ The experiment was approved by the Institutional Ethics Committee on Animal Use (516-2017) of Universidade Estadual Paulista, São Paulo, Brazil. Sample size estimates were based on data from the previous study. ^9 , 13 , 17^ Considering a 0.05 alpha error and 95% power to recognize a significant difference, seven animals were needed. Expecting animal attrition of 10%, the sample size calculated before was divided by 0.9, resulting in eight animals per group.

### Induction of AP

The rats were anesthetized via intramuscular injections of ketamine (87 mg/kg, Francotar; Virbac do Brazil Ind e Com Ltda, Roseira, São Paulo, Brazil) and xylazine (13 mg/kg, Rompum, Bayer AS, São Paulo, Brazil). Coronal pulp tissue was exposed and disorganized in the animals through an access cavity in the crown of the upper and lower first molar first molars using a spherical carbide bur with 0.5 mm diameter (Jet Carbide ¼, Kavo Kerr Group, Orange, CA, USA) with a surgical microscope (Decius, DF Vasconcelos, Rio de Janeiro, Brazil). The coronal pulp tissue was exposed and kept open to the oral cavity until euthanasia. ^16 , 17^

### Probiotic therapy

The animals were randomly assigned into two groups ( *n* = 8): rats with AP and fed with regular diet (CG); rats with AP, fed with regular diet and supplemented with GNC Probiotic Complex (PCG) ( *Lactobacillus acidophilus, Lactobacillus salivaris, Lactobacillus plantarum, Lactobacillus rhamnosus, Bifidobacterium bifidum, Bifidobacterium animalis subs. lactis* and *Streptococcus thermofilus* ) (CODE424639) (General Nutrition Centers, Inc., Nova York), United States. From the first day of apical periodontitis induction, PCG was orally administered via gavage. According to the manufacturer, one PCG capsule corresponds to one billion colony-forming units (CFU). Therefore, one capsule was diluted in 5 mL of water and administered daily to each animal in the PCG group during the development of apical periodontitis (30 days). The CG received 5 mL of water (without probiotics). ^18^

### Histopathological analyses

After 30 days, the animals were euthanized by an overdose of anesthetic solution. The maxilla and mandible were removed, fixed in a solution of 4% buffered formaldehyde for 24 h, and then decalcified in buffered (pH = 8) 17% EDTA (Sigma Chemical Co, St Louis, USA). Semi-serial 6 μm cuts were made from the upper and lower first molar and stained with hematoxylin and eosin (HE). Slides without staining were reserved for immunohistochemistry. Histopathological analyses using a light microscope (DM 4000 B; Leica) were performed by a certified histologist (E.E) who was blinded to the experimental groups and used a color camera (DFC 500; Leica, Wetzlar, Germany). The distal root was standardized for all analyses. ^17^ Histopathological analysis was conducted using the quality of inflammation and the cellularity pattern of dental and periodontal tissues as guidelines to score the inflammatory infiltrate as follows: absent (0 to few inflammatory cells, score = 1), mild (<25 cells, score=2), moderate (25–125 cells, score=3), and severe (>125 cells, score=4). ^19^ The presence or absence of necrosis was also recorded. ^9 , 17 , 20^

### Immunohistochemical analysis

Immunolabeling of the histological sections was performed using an indirect immunoperoxidase technique. ^9 , 17 , 20^ Histological sections were arranged into six batches, and each batch was incubated with one of the following primary antibodies (1:100): IL-10 (Rabbit - orb221323, Biorbyt, San Francisco, CA, USA); IL-1β (Rabbit - orb101745 Biorbyt), IL-6 (Rabbit - orb6210 Biorbyt), RANKL (Goat anti-RANKL - SC7627, Santa Cruz Biotechnology), OPG (Rabbit anti-OPG - SC11383, Santa Cruz Biotechnology) and TRAP (Goat anti-TRAP - SC30832, Santa Cruz Biotechnology, Santa Cruz, CA, USA). As a negative control, the specimens were subjected to the procedures described above by suppressing the primary antibody use.

Semiquantitative immunolabeling analyses of IL-10, IL-1β, IL-6, RANKL, and OPG were performed on the distal root apex by a certified histologist (E.E.), who was blinded to the experimental groups. ^17^ Three histologic sections were used for each animal, and positive immunoreactivity (IR) was defined as a brownish color in the cytoplasm of the cells and extracellular matrix. Because immunolabeling of both the cells and the extracellular matrix are of great importance for the study, a semiquantitative analysis was performed, providing information about the numbers of immunoreactive cells and immunolabeling intensity of the extracellular matrix. The scores were assigned as follows: 1=complete absence of immunoreactive cells; 2=low IR (a few immunoreactive cells and weak labeling of the extracellular matrix; approximately one-quarter of the immunoreactive cells); 3=moderate IR (a moderate number of immunoreactive cells and moderate labeling of the extracellular matrix; approximately half of the immunoreactive cells); and 4=high IR (a large number of immunoreactive cells and strong labeling of the extracellular matrix; approximately three-quarters of the immunoreactive cells). ^19^ The number of mature osteoclasts as TRAP-positive multinucleated cells was quantified in the perimeter of the lesion. The results were expressed as the number of multinucleated TRAP-positive cells per mm. ^20^

### Statistical Analysis

The data were analyzed using GraphPad Prism 7 software (La Jolla, CA, USA). After the Shapiro-Wilk test of normality, the Mann–Whitney test was performed for nonparametric data, and unpaired t-test was performed for parametric data. The level of significance was 5%.

## Results

**Figure 1:**
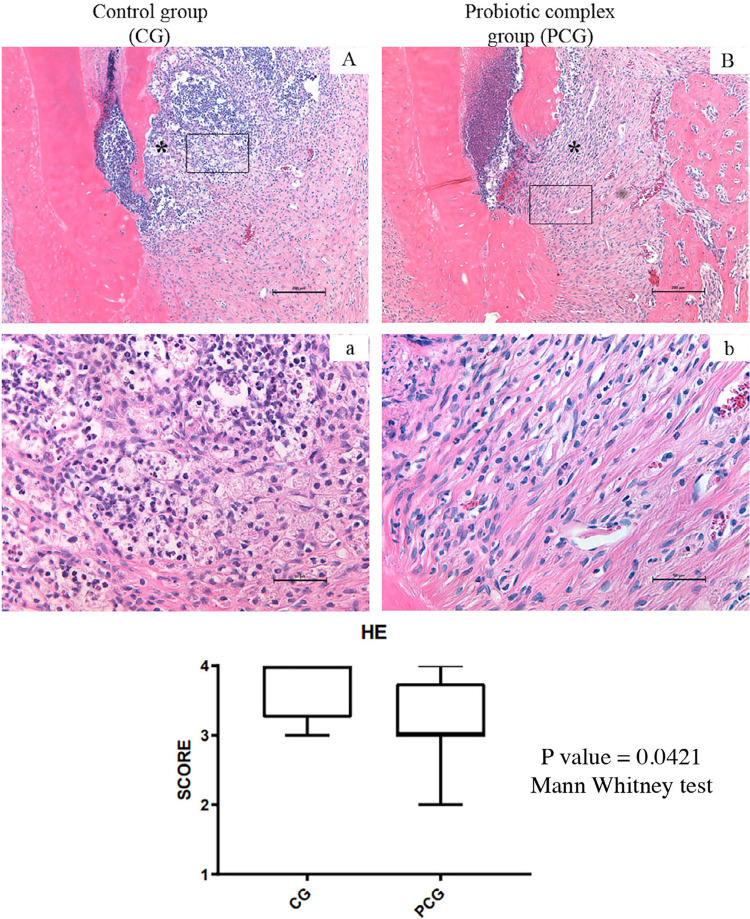
Representative photomicrographs showing histologic aspects of periapical regions (A-B, a-b). Control Group (n: 8) (A,a) showed more severe apical periodontitis than group that administered GNC Probiotic Complex (CODE424639) (n: 8). Haematoxylin–eosin staining. *Inflammatory infiltrate. Rectangle shows area elected for 400X magnification. Original magnification: A and B 100X; a and b 400X. Charts showing scores for inflammatory infiltrate

Figure 1 shows representative histologic images of AP in different experimental groups Necrosis was observed in the CG and PCG at 30 days after exposure. In AP, CG group showed more intense inflammatory infiltrates compared with that in the PCG group ( Figure 1 ). The median scores assigned to the CG group were significantly more severe than those in the PCG group *(P<.05)* .

**Figure 2:**
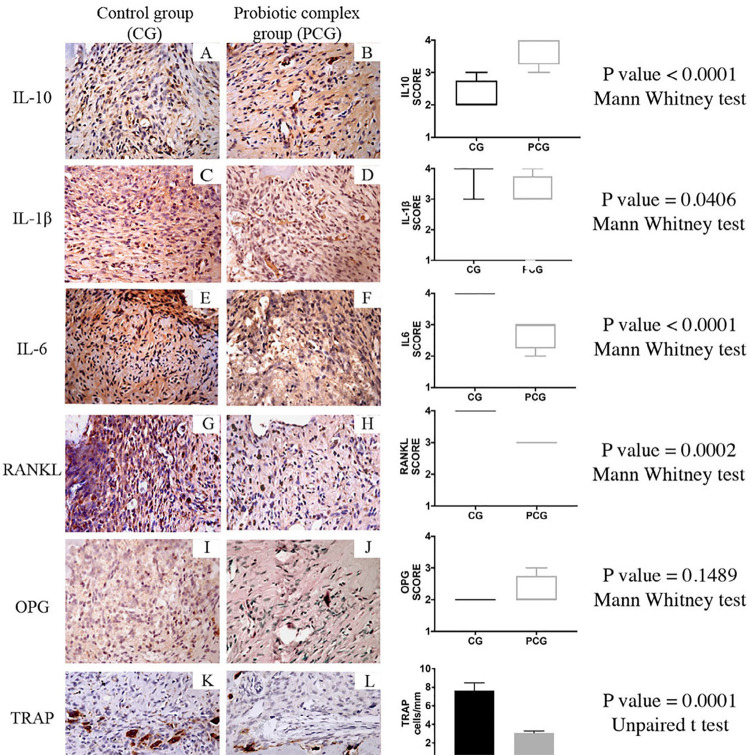
Representative photomicrographs showing immunolabeling of the periapical regions (A-L). IL-1β, IL 6 and RANKL decreased in the PCG compared with those in the CG; IL-10 level was increased in the PCG; OPG level was similar in CG and PCG; TRAP-positive multinucleated cells count per mm in the periapical region the PCG obtained lower count compared with the CG. Original magnification: 400X. Charts showing scores for IL-10, IL-1β, IL 6, RANKL, OPG and TRAP

Figure 2 shows representative immunohistochemistry images of AP in the CG and PCG groups. The levels of IL-1β, IL 6 and RANKL were significantly decreased in the PCG group compared with those in the CG group *(P<.05)* . The IL-10 level was significantly increased in the PCG group *(P<.05)* . The OPG level was similar in both groups *(P>.05)* . The PCG group showed significantly lower TRAP-positive multinucleated cells count in the periapical region when compared to the C group *(P<.05).*

## Discussion

This is the first study in which a multi-strain probiotics formula is administered to evaluate its effect on the apical periodontitis development in rats. The null hypothesis was rejected once the pathogenic inflammation/bone resorption significantly diminished when systemic supplementation with probiotic complex was used. Previous studies have shown that *Lactobacillus acidophilus* and *Lactobacillus rhamnosus* , administered separately, affected the apical periodontitis development. ^9 , 13^ In our study, the use of a multi-strain probiotics formula led to less intense inflammation and also a smaller number of osteoclast cells when compared to previous studies that used the same methodology. Therefore, the use of a multi-strain probiotics formula may stand as an alternative in decreasing the intensity of inflammatory process and bone resorption in apical periodontitis.

No single probiotic bacterium exhibits all the desirable properties. A combination of different strains might be used to maximize their beneficial effects. ^21^ The probiotic complex used in this study was composed of the following strains: *Lactobacillus acidophilus, Lactobacillus salivaris, Lactobacillus plantarum, Lactobacillus rhamnosus, Bifidobacterium bifidum, Bifidobacterium animalis subs. lactis* and *Streptococcus thermofilus* . The effectiveness of multi-strain probiotics has been previously evaluated in periodontitis patients. ^22^ This study showed improvement in immunologic response by a reduction in matrix metalloproteinase-8 levels in gingival crevicular fluid in chronic periodontitis patients; ^22^ depth reduction in moderate pockets; and reduced oral malodor in patients with chronic periodontitis and halitosis. ^23^

In our study, the AP model employed is supported by the scientific literature, and the authors have extensive practice with their management. ^9 , 13 , 16 , 17 , 20^ The apical periodontitis induction in rats allows researchers to detach extrinsic factors that would be difficult to manage if the investigation was performed as a clinical study. ^16 , 17 , 19 , 20 , 24^

The probiotics can kill or inhibit the bacteria, modify the host immunity by reduction of pro-inflammatory and elevation of anti-inflammatory cytokines, as well as modify the cell proliferation and apoptosis. ^25^ In this study, diminished expression of IL-1β and enhanced expression of IL-10 were observed in PCG when compared with CG. IL-10 can inhibit IL-1β and tumor necrosis factor-α, which present synergistic actions on inflammatory processes, amplifying the host response. ^26^ The increased ratio of IL-1β/IL-10 is a relevant progression marker of periapical periodontitis. ^9^ The *Lactobacillus acidophilus* present in the complex has been previously reported to influence one or more components of humoral, cellular, or activate nonspecific immunity. ^8^ Moreover, it can upregulate IL-10 and downregulate IL-1β. ^10 , 11^ *Bifidobacterium bifidum* can increase levels of IL-10, amplifying the host response. ^3^

*Streptococcus thermophilus* present in the probiotic complex is also a potent inducer of the anti-inflammatory IL-10 in macrophages. ^27^ Furthermore, this strain can produce thermophilins called bacteriocins, which are proteins produced by certain bacteria, having a characteristic feature of inhibiting the growth of similar or closely related bacterial strains. Thermophilins have been shown to have *in vitro* inhibitory activities against Gram-positive pathogenic strains such as *E. faecalis* , one of the most challenging bacterial species to eliminate in the development of apical periodontitis. ^28^

A reduced number of TRAP-positive multinucleated cells was found in PCG when compared with CG. This may be partially explained by the reduced RANKL immunostaining present in PCG. RANKL is the positive regulator for osteoclastogenesis and OPG acts as an inhibitory factor, binding to RANK and preventing RANKL from binding to it. ^29 , 30^ In this way, it prevents the sequence of fundamental events to the osteoclast’s formation. In our study, the OPG values remained stable in both groups and the RANKL decreased in the PCG group, explaining the low TRAP-positive multinucleated cells.

*Lactobacillus plantarum* has immunoregulatory function through activation of Th1 immune responses, promotion of IgA secretion and improvement of natural killer (NK) cell activity. ^31^ *Lactobacillus rhamnosus* is related to increased levels of peripheral blood, polymorphonuclear leukocytes cell response and natural killer cell activity, presenting an immunomodulatory action by regulating cytokines as TNF- alpha, IL-6, IL-12 and IL-10. ^9 , 32^ *Bifidobacterium animalis subs. lactis* exerts anti-inflammatory effects on the epithelium by down-regulating the secretion of IL-8, which is considered a potential probiotic because it possesses immunomodulatory and antimicrobial properties. ^18^

Probiotics stimulate dendritic cells (antigen-presenting cells), resulting in the expression of T-helper cell 1 (Th1) or Th2 response, which will phagocyte intracellular and extracellular pathogens respectively, modulating the immune system. ^33^ Probiotics enhance innate immunity and modulate pathogen-induced inflammation through Toll-like receptors on dendritic cells. ^33^ Intracellular pathogens are phagocytosed by Th1 response, and extracellular pathogens by the Th2 response. ^33^ In fact, the rationale for probiotics use is mainly based on their ability to promote growth and survival of commensal bacteria. ^34^

Our study yielded important information on the therapeutic potential of probiotic in apical periodontitis. Clinically, these results can be considered a potentially promising option (combined with the traditional one) in the treatment of apical periodontitis, since the probiotics reduced the severity of the inflammation, what could result in a faster apical repair process. However, the results should not be directly translated to humans. It is important to remember that probiotic findings cannot be generalized since they are dependent on strain, dosage, frequency, and experimental model. Besides, randomized clinical studies should be conducted to better elucidate the use of probiotics in the treatment of apical periodontitis.

## Conclusion

Supplementation with multi-strain probiotics formula had a significant effect on reducing inflammation and bone resorption in apical periodontitis.
